# Supplementary material for: ProteinShader: illustrative rendering of macromolecules
Source: BMC Struct Biol. 2009 Mar 30;9:19. doi: 10.1186/1472-6807-9-19 (PMC2672931; doi:10.1186/1472-6807-9-19)
Supplement: Additional file 1 — ProteinShader program without source code. This compressed file contains the complete ProteinShader program including associated libraries, but no source code. A README.txt file gives an overview of the ProteinShader distribution, and the index.html file in the help subdirectory has directions on getting started with the program as well as a set of tutorials. [file 1472-6807-9-19-S1.zip › ProteinShader-beta-0_9_4-binary/help/api/org/proteinshader/gui/listeners/menubar/BackgroundMenuListenerFactory.html]

BackgroundMenuListenerFactory (ProteinShader API)


|  |  |  |
| --- | --- | --- |
| **Overview** \| **Package** \| **Class** \| **Use** \| **Tree** \| **Deprecated** \| **Index** \| **Help** | |  |
| **PREV CLASS**   **NEXT CLASS** | **FRAMES**    **NO FRAMES**     **All Classes** |  |
| SUMMARY: NESTED \| FIELD \| CONSTR \| METHOD | DETAIL: FIELD \| CONSTR \| METHOD |  |


---


## org.proteinshader.gui.listeners.menubar Class BackgroundMenuListenerFactory

```
java.lang.Object
  org.proteinshader.gui.listeners.menubar.BackgroundMenuListenerFactory
```

---

``` public class BackgroundMenuListenerFactory extends Object ```

Creates the event listeners needed by the BackgoundMenu of the MainMenuBar.

---

**Constructor Summary**

`BackgroundMenuListenerFactory(Mediator mediator,
BackgroundMenu backgroundMenu)`
  
          Constructs a BackgroundMenuListenerFactory.


| **Method Summary** | |
| --- | --- |
| `ActionListener` | `creatCustomMenuItemListener()`             Creates an action listener for the 'Custom' menu items. |
| `ActionListener` | `createBlackMenuItemListener()`             Creates an action listener for the 'Black' menu items. |
| `ChangeListener` | `createChooserChangeListener()`             Creates a ChangeListener that will get the color selection from the JColorChooser and calls on the Mediator to change the background color of the canvas. |
| `ActionListener` | `createGrayMenuItemListener()`             Creates an action listener for the 'Gray' menu items. |
| `ActionListener` | `createLightGrayMenuItemListener()`             Creates an action listener for the 'Light Gray' menu items. |
| `ActionListener` | `createWhiteMenuItemListener()`             Creates an action listener for the 'White' menu items. |

**Methods inherited from class java.lang.Object**

`clone, equals, finalize, getClass, hashCode, notify, notifyAll, toString, wait, wait, wait`

**Constructor Detail**

### BackgroundMenuListenerFactory

```
public BackgroundMenuListenerFactory(Mediator mediator,
                                     BackgroundMenu backgroundMenu)
```

:   Constructs a BackgroundMenuListenerFactory.

    **Parameters:**: `mediator` - the centralized Mediator object that listeners call on to accomplish their task.


**Method Detail**

### createBlackMenuItemListener

```
public ActionListener createBlackMenuItemListener()
```

:   Creates an action listener for the 'Black' menu items. When
    the action listener is called, it will call on the Mediator to
    have the Renderer set the background color of the canvas to black.

    :   **Returns:**: The action listener.

---


### createGrayMenuItemListener

```
public ActionListener createGrayMenuItemListener()
```

:   Creates an action listener for the 'Gray' menu items. When
    the action listener is called, it will call on the Mediator to
    have the Renderer set the background color of the canvas to gray.

    :   **Returns:**: The action listener.

---


### createLightGrayMenuItemListener

```
public ActionListener createLightGrayMenuItemListener()
```

:   Creates an action listener for the 'Light Gray' menu items. When
    the action listener is called, it will call on the Mediator to
    have the Renderer set the background color of the canvas to light
    gray.

    :   **Returns:**: The action listener.

---


### createWhiteMenuItemListener

```
public ActionListener createWhiteMenuItemListener()
```

:   Creates an action listener for the 'White' menu items. When
    the action listener is called, it will call on the Mediator to
    have the Renderer set the background color of the canvas to white.

    :   **Returns:**: The action listener.

---


### creatCustomMenuItemListener

```
public ActionListener creatCustomMenuItemListener()
```

:   Creates an action listener for the 'Custom' menu items. When
    the action listener is called, it will call on the BackgroundMenu
    to open a JColorChooser in a dialog box.

    :   **Returns:**: The action listener.

---


### createChooserChangeListener

```
public ChangeListener createChooserChangeListener()
```

:   Creates a ChangeListener that will get the color selection from
    the JColorChooser and calls on the Mediator to change the
    background color of the canvas.

    :   **Returns:**: The action listener.


---


|  |  |  |
| --- | --- | --- |
| **Overview** \| **Package** \| **Class** \| **Use** \| **Tree** \| **Deprecated** \| **Index** \| **Help** | |  |
| **PREV CLASS**   **NEXT CLASS** | **FRAMES**    **NO FRAMES**     **All Classes** |  |
| SUMMARY: NESTED \| FIELD \| CONSTR \| METHOD | DETAIL: FIELD \| CONSTR \| METHOD |  |


---

# *Copyright © 2007-2008*
